# Supplementary material for: Protocol for a prospective observational cohort study to assess clinical applications of expanded noninvasive prenatal testing (NIPT) in pregnancies with placental dysfunction
Source: PLoS One. 2026 Apr 9;21(4):e0344772. doi: 10.1371/journal.pone.0344772 (PMC13065038; doi:10.1371/journal.pone.0344772)
Supplement: S1 Appendix — (DOCX) [file pone.0344772.s001.docx]

**Appendix: Description of study variables and data collection**

*Principal variable*

- Detection of RATs and CNVs ≥7 Mb detection by expanded NIPT [yes/no]

*Secondary variables*

Descriptive variables:

- Expected date of delivery [date]

- Expected date of delivery based on [last menstrual period, crown rump length measurement, date of fertilization in case of assisted reproduction]

- Maternal age at delivery [years]

- Maternal weight at the start of pregnancy [kg]

- Maternal height [cm]

- Nicotine use during pregnancy [cigarettes/day]

- Alcohol consumption during pregnancy [units consumed]

- Substance abuse during pregnancy [yes/no]

- Type(s) of drugs used [none, cannabis, cocaine, amphetamines, opiates, other]

- Ethnicity/self-perceived race [Caucasian, Asian, North African, South American, African American, other]

- Level of education achieved [none, primary, incomplete secondary, complete secondary, incomplete university degree, bachelor’s degree, master’s degree, doctoral degree]

- Method of conception [spontaneous, artificial insemination, in vitro fertilization, egg donation]

- Number of previous pregnancies [continuous]

- Number of previous vaginal deliveries [continuous]

- Number of previous caesarean sections [continuous]

- Number of previous intrauterine deaths [continuous]

- Number of previous spontaneous miscarriages [continuous]

- Number of previous terminations of pregnancy [continuous]

- FGR during previous pregnancy [yes/no]

- Pre-eclampsia during previous pregnancy [yes/no]

- Chronic hypertension [yes/no]

- Chronic kidney disease [yes/no]

- Pre-pregnancy diabetes [yes/no]

- Prior pregnancies with chromosomal abnormalities [yes/no]

- Known familiar genetic disease [yes/no]

- Previous genetic tests [yes/no]

- Thrombophilia [yes/no]

- Systemic lupus erythematosus [yes/no]

- Antiplatelet medication [no, yes started ≤16 weeks of gestation, yes started >16 weeks of gestation]

- Anticoagulant medication [no, yes started ≤16 weeks of gestation, yes started >16 weeks of gestation]

-Corticosteroids [yes/no]

- Ultrasound parameters obtained at diagnosis of eoFGR:

- Mean uterine artery pulsatility index adjusted for gestational age [continuous]

- Estimated fetal weight [g]

- Estimated fetal weight percentile [continuous]

- Umbilical artery PI, middle cerebral artery PI, cerebroplacental ratio (middle cerebral artery PI: umbilical artery PI), ductus venosus PI if CPR <10th percentile [continuous and percentile]

Laboratory/genetic variables:

- PAPP-A (MoM) [continuous]

- β-hCG (MoM) [continuous]

- Gestational age at PAPP-A and β-hCG measurement [weeks]

- Describe which laboratory platform is used for PAPP-A and b-hCG determinations [free text]

- High risk for fetal aneuploidy (>1:270 for trisomy 21 or trisomy 18) [yes/no]

- sFlt-1:PlGF ratio at eoFGR diagnosis [continuous]

- Prenatal genetic testing performed [none, targeted NIPT, expanded NIPT, CVS, Amniocentesis (AC)]

- Type of genetic test done when invasive testing [QF-PCR, karyotype in amniotic fluid, karyotype in short term culture (STC) chorionic villi, karyotype in long term culture (LTC) chorionic villi, CGH array, SNP array]; cytogenomic test results are noted according to the 2024 edition of the International System for Human Cytogenomic Nomenclature (ISCN 2024)

- Genetic alterations detected by targeted NIPT [none, trisomy 21, trisomy 18, trisomy 13, monosomy X, other]

- Genetic alterations detected by expanded NIPT [none, trisomy 21, trisomy 18, trisomy 13, monosomy X, RAT, CNV, other]

- Genetic alterations detected by invasive prenatal testing [n/a, none, trisomy 21, trisomy 18, trisomy 13, monosomy X, RAT, CNV, other]

- Description of other genetic alterations

- Genetic alterations detected in placental biopsies [n/a, none, trisomy 21, trisomy 18, trisomy 13, monosomy X, RAT, CNV, other]

- Genetic alterations detected in newborn [n/a, none, trisomy 21, trisomy 18, trisomy 13, monosomy X, RAT, CNV, other, describe tissue/blood]

- Placental histopathological findings [n/a, none, maternal hypoperfusion, fetal hypoperfusion, both, describe findings/conclusion] (free text) [23]

- Fetal fraction [%]

- Mosaic ratio [%]

- Aneuploidy log-likelihood ratio score [continuous]

- Mosaic/Default log-likelihood ratio score [continuous]

Result variables:

- Major malformation [yes/no]

- Description of major malformation [free text]

- Gestational hypertension; de novo preeclampsia, superimposed preeclampsia according to the definitions of the International Society for the Study of Hypertension in Pregnancy (ISSHP) [24] [yes/no with description]

- EoFGR [yes/no]

- Termination of pregnancy [yes/no]

- Late miscarriage (up to 22 weeks) [yes/no]

- Intrauterine death (greater than 22 weeks) [yes/no]

- Neonatal death [yes/no]

- Gestational age at birth [weeks + days]

- Type of delivery [spontaneous, instrumental, caesarean section on foetal indication, caesarean section for other reasons]

- Neonatal weight [g and percentile]

- Sex of newborn [female, male]

- Neonatal acidosis (arterial pH <7.10 or base excess >12mEq/l) [yes/no]

- Apgar score <7 at 5 minutes [yes/no]

- Suspected neonatal infection [no, yes (not confirmed), yes (confirmed with positive culture)]

- Respiratory distress [yes/no]

- Transient tachypnoea of the newborn [yes/no]

- Meconium aspiration syndrome [yes/no]

- Hypoglycaemia [yes/no]

- Perinatal asphyxia [yes/no]

- Admission to the Neonatal Intensive Care Unit (NICU) [yes/no]

- Duration of NICU admission [days]

- Genetic abnormality detected postpartum [yes/no]

- Description of genetic abnormality detected postpartum [free text]

- HPO phenotype [free text]

-Abnormal outcome at follow-up (up to age of six months) [yes/no]
